# An Increase in Phosphorylation and Truncation of Crystallin With the Progression of Cataracts^☆^

**DOI:** 10.1016/j.curtheres.2012.10.003

**Published:** 2013-06

**Authors:** Hui-Ju Lin, Chien-Chen Lai, Shiuan-Yi Huang, Wei-Yi Hsu, Fuu-Jen Tsai

**Affiliations:** 1 Department of Ophthalmology, China Medical University Hospital, Taichung, Taiwan; 2 Department of Medical Science, China Medical University Hospital, Taichung, Taiwan; 3 Department of Chinese Medicine, China Medical University, Taichung, Taiwan; 4 Institute of Molecular Biology, National Chung Hsing University, Taichung, Taiwan

**Keywords:** cataract, crystallin, mass spectrometry, phosphorylation, truncation

## Abstract

**Background:**

Cataracts are the leading cause of blindness worldwide; however, there is no evidence regarding the direct formation of cataracts. At present, there is no treatment method other than surgery to prevent the formation or progression of cataracts.

**Objective:**

Understanding the protein changes during various stages of cataracts might help realize the mechanism of the formation and progression of cataracts.

**Methods:**

Lens materials were collected from cataract surgery. Cataracts were classified according to lens opacity using the gradation of the Lens Opacities Classification System. Lens proteins were separated by 2-dimensional polyacrylamide gel electrophoresis. Protein spots were visualized by Coomassie blue staining, and expression patterns were analyzed. Protein spots of interest were excised from 2-dimensional polyacrylamide gel electrophoresis gels, digested in situ with trypsin, and analyzed by mass spectrometry and liquid chromatographic tandem mass spectrometry.

**Results:**

Crystallin was the major protein in the cataract lens, and αA, βB1, αB, and βA4 were the dominant types. Crystallin αB and βA4 increased with the formation of lens opacity. Moreover, phosphorylation and truncation of these proteins increased with the progression of cataracts.

**Conclusion:**

Crystallin αB and βA4 and phosphorylation and truncation of crystallin in the lens might contribute to the formation of cataracts. In contrast, acetylation was not dominant in the progression of cataracts and did not play major role in the formation of cataracts.

## Introduction

The effect of cataracts on vision is often described as being similar to looking through a waterfall or a piece of waxed paper. Poor vision from cataracts affects 80% of people aged ≥75 years.^1^ This disease causes clouding of the eye lens, which reduces the amount of incoming light and deteriorates vision. Daily functions such as reading or driving a car may become difficult or impossible.^2^Thus, patients may require frequent change in eyeglass prescriptions.^3^ It is estimated that 200 million people have cataracts worldwide.^4,5^ Data from the National Institutes of Health indicate that >350,000 cataract surgeries are performed annually in the United States.

The lens is the clear part of the eye that helps to focus light and images on the retina.^6^ The retina is the light-sensitive tissue at the back of the eye. In a normal eye, light passes through the transparent lens to the retina. Once it reaches the retina, light is changed into nerve signals that are sent to the brain.^7^ The lens must be clear for the retina to receive a sharp image. If the lens is cloudy as a result of cataracts, the image will be blurred. The lens is mostly made of water and protein. The proteins are arranged to let light pass through and focus on the retina. Sometimes, some lens proteins clump together and begin to cloud a small area of the lens. Over time, the cells accumulate and cause the lens to cloud, thereby resulting in blurred or fuzzy images.^4,8^ Cataracts are the leading cause of visual loss among adults >55 years old. Cataract surgery costs Medicare more money than any other medical procedure, with 60% of those who initially qualify for Medicare already having cataracts.^9^ Most people are concerned regarding the time of onset of cataracts and not about its occurrence. Hence, preventative steps at an early stage in life may lead to good eye health and prevent cataracts.^10^ Many factors influence vision and cataract development, for example, age, nutrition, heredity, medications, toxins, health habits, sunlight exposure, and head trauma. Cataracts can also be caused by high blood pressure, kidney disease, diabetes, or direct trauma to the eye.^11,12^ Although cataract surgeries have advanced progressively, cataracts are still the leading cause of blindness and are a profound economic cost to society.^13–15^ In this study, we used 2-dimensional polyacrylamide gel electrophoresis (2D-PAGE) to identify the proteins that change during the formation of lens opacity and liquid chromatographic tandem mass spectrometry (LC/MS/MS) to evaluate post-translation modifications of the proteins. Investigating the protein changes during various stages of cataracts elucidated the mechanism of the formation of cataracts and might be helpful in designing new therapies.

## Materials and Methods

Lens materials were obtained during cataract surgery. All patients in this study received serial ophthalmic examinations, including intraocular pressure (IOP), visual acuity, and retinal examination. Patients with ocular diseases other than cataracts were excluded. Patients (38 women and 42 men), aged 56 to 85 years (mean, 72 years), were followed up after 3 to 24 weeks (mean, 5 weeks). The study was performed according to the tenets of the Declaration of Helsinki for research involving human participants.

Patients with Stage 2 to 5 cataracts were enrolled in the study. The patients did not have any systemic diseases or eye diseases other than cataracts. They underwent phacoemulsification surgery using a phacoemulsification machine (Universal II; Alcon, Houston, Texas). Lens materials were collected into bags by the machine after quaking the lens into small particles by ultrasound. Lens opacity was classified according to the Lens Opacities Classification System (LOCS) before surgery.^16,17^ This classification involves comparison of the slit lamp view of the lens to a color plate of LOCS III standards. LOCS uses standard reference photographs taken during slit lamp examination. The extent of opacification of cortical (C) and posterior subcapsular (P) changes was defined, and color changes of the nucleus as well as the intensity of nuclear opalescence was noted using LOCS. Lens opacity was scored according to the description in LOCS, for example, N0 and NIV were denoted as 0 and 4, respectively. After adding the scores of the 4 parts (nuclear color, nuclear opacity, cortical cataracts, and posterior subcapsule), a total score (0–15) was obtained. These scores were used to categorize cataracts into 5 stages: score 0, Stage 1; scores 1 to 4, Stage 2; scores 5 to 8, Stage 3; scores 9 to 12, Stage 4; and scores 13 to 15, Stage 5.

## Two-dimensional Gel Electrophoresis

### Sample Preparation and Running of Gels

An aliquot containing 100 μg of protein sample was diluted with 350 μL of rehydration buffer containing 8-M urea, 4% [3-(3-cholamidopropyl)dimethylammonio]-1- propanesulfonate], 65-mM dithioerythritol (DTE), 0.5% ampholytes, and a trace of bromophenol blue. An immobilized pH gradient (17 cm; pH 3–10; ReadyStrip IPG strip; Bio-Rad, Tokyo, Japan) was hydrated overnight, and the samples were focused for a total of 60 kVh (PROTEAN IEF cell; Bio-Rad) at 20°C and then stored at −80°C. Strips were equilibrated with 3 mL of an equilibrium solution containing 50-mM Tris–hydrocholride (pH 8.8), 6-M urea, 30% glycerol, 2% sodium dodecyl sulfate (SDS), a trace of bromophenol blue, and DTE (1% w/v) for 20 minutes, followed by equilibration for 20 minutes in the same solution containing iodoacetamide (IAA; 2.5% w/v) instead of DTE. The strips were transferred to the tops of 12% PAGE and held in position with molten 0.5% agarose in running buffer containing 25-mM Tris, 0.192-M glycine, and 0.1% SDS. The gels were run at 16 mA for 30 minutes, followed by 50 mA for 4 to 5 hours.

### Detection of Protein Spots and Data Analysis

The gels were routinely stained with Coomassie blue and then scanned using a GS-800 imaging densitometer with PDQuest software (version 7.1.1; Bio-Rad). To evaluate intra- and intersample variability, the gels were analyzed as follows: protein spots from each gel were detected and matched automatically to generate a master gel image from the matched gel sets. Finally, the intensity of the spots was compared between gels. Data were exported to Microsoft Excel (Microsoft Inc, Redmond, Washington) for creating correction and spot intensity graphs.

### In-Gel Digestion

The procedure of Terry et al^18^ was slightly modified and used for in-gel digestion of proteins from the Coomassie blue-stained gels for nanoscale capillary LC/MS/MS. In brief, each spot of interest on the Coomassie blue-stained gel was sliced into 1-mm cubes. The proteins in these gels were reduced and methylated with 50-mM DTE and 100-mM IAA in 50-mM ammonium bicarbonate. The gel pieces were washed 2 times with 50% v/v acetonitrile (ACN) in 100-mM ammonium bicarbonate buffer (pH 8.0) for 10 minutes at room temperature. They were then soaked in 100% ACN for 5 minutes, dried in a lyophilizer for 20 to 30 minutes, and rehydrated in 50-mM ammonium bicarbonate buffer (pH 8.0) containing 10 μg/mL trypsin (Promega, Madison, Wisconsin) until fully immersed. After incubating for 16 to 20 hours at 30°C, the remaining trypsin solution was transferred into a new microtube. The gel pieces were resuspended with 50% ACN in 5.0% formic acid (FA) for 60 minutes, and then concentrated to dryness.

## Nanoelectrospray Mass Spectrometry

Nanoscale capillary LC/MS/MS was used to analyze the proteins involved in the development of cataracts. The Ultimate Capillary LC System (LC Packings, Amsterdam, the Netherlands) coupled to a QSTARXL quadrupole-time of flight (Q-TOF) mass spectrometer (Applied Biosystem/MDS Sciex, Foster City, California) was used for analysis. Nanoscale capillary LC separation was performed on a reverse phase C18 column (15 cm × 75 μm inner diameter) with a flow rate of 200 nL/min and a 60-minute linear gradient of 5% to 50% buffer B. Buffer A contained 0.1% FA in 5% aqueous ACN, and buffer B contained 0.1% FA in 95% aqueous ACN. The nano-LC tip for online LC/MS was a PicoTip (FS360-20-10-D-20; New Objective, Cambridge, Massachusetts). Data acquisition was performed using automatic information dependent acquisition (IDA; Applied Biosystem/MDS Sciex). Automatic IDA finds the most intense ions in TOF MS spectra and then performs optimized MS/MS analysis on these ions. The product ion spectra generated by nano-LC/MS/MS were searched against National Center for Biotechnology Information (NCBI) databases for exact matches using the ProID program (Applied Biosystem/MDS Sciex) and the MASCOT search program (MASCOT search program; Matrix Science, Inc, Boston, Massachusetts). A mammalian taxonomy restriction was used, and the mass tolerance of both precursor and fragment ions was set to ± 0.3 Da. Carbamidomethyl cysteine was set as a fixed modification, whereas phosphorylation of serine, threonine, and tyrosine, and other modifications were set as variable modifications. All identified phosphopeptides were confirmed by manual interpretation of the spectra.

## Results

**Figure 1:**
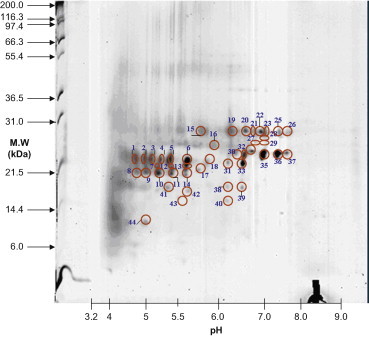
Spots 1 to 44 existed in Stage 2 cataracts. MW, molecular weight.

**Figure 2:**
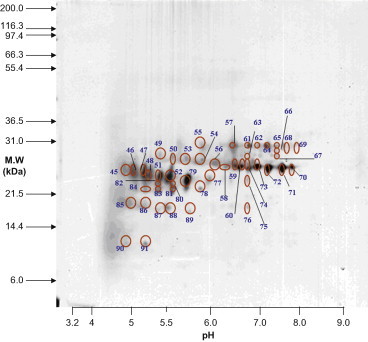
Spots 45 to 91 existed in Stage 3 cataracts. MW, molecular weight.

**Figure 3:**
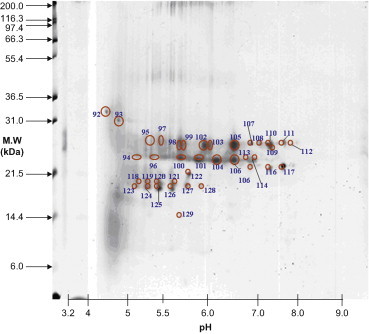
Spots 92 to 129 existed in Stage 4 cataracts. MW, molecular weight.

**Figure 4:**
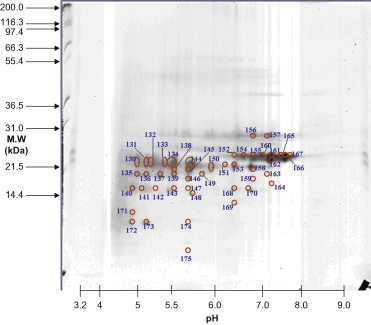
Spots 130 to 175 existed in Stage 5 cataracts. MW, molecular weight.

Lens materials from various stages were prepared for 2D-PAGE. To identify protein expression, master gels were computed from scanned images of quartet silver-stained gels. The scanned gel images were processed using the Proteomics Software System developed by Xzillion (Frankfurt am Main, Germany). The master gel was computed by registering and jointly segmenting multiple registered replicates. Algorithmic details can be found in the SEQUEST algorithm (version C1; Thermo Fisher Scientific, Waltham, Massachusetts) incorporated into the ThermoFinnigan BIOWORKS software (version 3.0; Thermo Fisher Scientific). Spot volumes were determined by modeling optical density of individual spot segments using 2D Gaussian analysis. To correct for variability due to gel electrophoresis, quartet gels were run for each cataract stage. In addition, spots expressed in <50% of the gels were disregarded. Furthermore, upregulation of proteins was considered significant when the corresponding spot volumes were increased by more than twofold. Representative master gels showed proteins expressed in different stages of cataracts. The proteins varied between 10 and 120 kDa in size and had isoelectric point (pI) values ranging from 5 to 9 (Figures 1–4).

No patient with Stage 1 cataracts underwent surgery; therefore, data were collected from patients with Stage 2 to 5 cataracts. Spots 1 to 44 were expressed in Stage 2 (Figure 1 and Tables I and II). After analysis, the major proteins in Stage 2 were crystallin βB1, αB, α A, and βA4. Eleven spots were identified as crystallin βB1. Of these, 5 were phosphorylated, 1 was acetylated, and none was truncated. In addition, 11 protein spots were identified as crystallin αB in Stage 2. Of these, 5 were phosphorylated, 1 was both phosphorylated and acetylated, and none was truncated. Eleven spots were identified as crystallin αA. Six of these were phosphorylated, 6 were acetylated, 5 were both phosphorylated and acetylated, and all were truncated. Eight spots were identified as crystallin βA4. Of these, 3 were phosphorylated and none was acetylated. However, all crystallin βA4 proteins were truncated. Among the protein spots in Stage 2, 46.3% were phosphorylated (Tables I and II); the ratio of phosphorylated to nonphosphorylated proteins was 17:24 (41.46% phosphorylated proteins). Acetylated proteins were more abundant than nonacetylated ones; the ratio of acetylated to nonacetylated proteins was 8:33. Only 19.5% of crystallin proteins in Stage 2 were acetylated. The ratio of truncated to nontruncated proteins was 19:22 (46.3% truncated) (Tables I and II). The proportions of phosphorylated and truncated proteins were high in Stage 2, whereas that of acetylated proteins was low.

The major proteins associated with Stage 3 (Figure 2 and Tables I and II) were crystallin αA, βB1, βA3, and βA4. Thirteen proteins were identified as crystallin αA, 6 of which were phosphorylated, 7 were acetylated, and 5 were both phosphorylated and acetylated. All crystallin αA proteins in Stage 3 were truncated. Ten proteins were identified as crystallin βB1. Of these, 8 were phosphorylated, 2 were acetylated, and none was truncated. Six proteins were identified as crystallin βA3. Of these, 5 were phosphorylated, 1 was acetylated, and all 6 were truncated. Six proteins in Stage 3 were identified as crystallin βA4. None of these were phosphorylated or acetylated; however, they were all truncated. In Stage 3, the ratio of phosphorylated to nonphosphorylated proteins was 19:16 (54.2% phosphorylated). The ratio of acetylation was lower than that of phosphorylation, with 10:25 (28.6%) proteins carrying acetyl groups (Tables I and II). The ratio of truncated to nontruncated proteins was 25:10, indicating that more than half of the proteins in Stage 3 were truncated (71.4%; Tables I and II). The phosphorylation ratio in Stage 3 was higher than that in Stage 2 (Stage 3 to Stage 2, 54.3%:46.3%). Similarly, the acetylation ratio in Stage 3 was also higher than that in Stage 2 (Stage 3 to Stage 2, 28.6%:19.5%). In contrast, the truncation ratio was increased in Stage 3 (Stage 3 to Stage 2, 71.4%:46.3%) (Tables I and II).

The major lens proteins associated with Stage 4 were crystallin βB1 and βA4 (Figure 3 and Table II). Sixteen proteins were identified as crystallin βB1. Of these, 4 were phosphorylated, and none was acetylated or truncated. In addition, 9 proteins were identified as crystallin βA4, none of which was modified or truncated. The ratio of phosphorylated to nonphosphorylated proteins in Stage 4 was 4:21. The prevalence of modifications such as phosphorylation (16%), acetylation (0%), and truncation (0%) decreased in Stage 4 (Tables I and II).

The major protein components associated with Stage 5 cataract lenses (Figure 4 and Tables I and II) were crystallin αA, βA4, and αB. Of these, 19 proteins were identified as truncated crystallin αA, and 11 of them were phosphorylated. Fourteen proteins were identified as crystallin αB. Of these, 8 were phosphorylated, 4 were acetylated, and none was truncated. Six proteins were identified as crystallin βA4, 1 of which was phosphorylated, and none was acetylated or truncated. In Stage 5, the ratio of phosphorylated to nonphosphorylated proteins was 20:19, that of acetylated to nonacetylated proteins was 4:35, and that of truncated to nontruncated proteins was 19:20. The prevalence of phosphorylation (51.3%) and truncation (48.7%) increased again in Stage 5, whereas that of acetylated crystallin proteins (10.3%) remained low (Table II).

## Discussion

Cataract and intraocular lens surgery is progressing at an astonishing speed.^19,20^ Nevertheless, cataracts are still the leading cause of blindness worldwide,^4,5,13^ especially in underdeveloped countries where cataract surgery is not widely available.^15^ Where it is available, cataract surgery continues to be expensive, representing a significant cost to health services in many nations.^21^ The etiology of cataracts involves induction of free radicals and superoxide-mediated damage to lens proteins by ultraviolet (UV) light.^22,23^ The mechanisms of this disease remain elusive, and preventative medicines have not yet been discovered.^20^ In this study, we used proteomic analyses to determine differential expression and post-translational modifications of lens proteins during the development of cataracts.

Crystallins were identified as the most differentially expressed proteins in cataract lenses. The pI of human lens proteins was distributed from 5 to 9, and the molecular weight was between 10 and 120 kDa. Phosphorylation and truncation were increased in the early stages of lens clouding, indicating that these modifications of crystallins might contribute to the formation of cataracts.^24–27^ Acetylation of crystallins was not as marked as phosphorylation in the opaque lens, although acetylation increased with the progression of cataracts. In contrast, phosphorylation, truncation, and acetylation decreased in Stage 4. This might indicate that the modification of crystallin proteins is not important in maintaining lens opacity in late-stage cataracts. In addition, microscopic structures of lenses begin to deteriorate in Stage 4; however, modification and truncation of crystallin proteins were not predominant in this stage. The major components of human lens crystallin were αA, βB1, αB, and βA4. The abundance of crystallin βA4 did not change with the progression of cataracts, and it remained the major component in every stage. Crystallin βB1 was also a dominant component in Stages 2 to 4 human cataract lenses; however, it disappeared in Stage 5 cataracts. These data suggested that crystallin βB1 is important in the progression of lens opacity in early and middle stages. Nevertheless, in severely opaque lenses, such as those in Stage 5, the basic structure had deteriorated to that of severe cataracts, such as morgagnian cataracts, and crystallin βB1 was totally absent. Crystallin αA was predominantly expressed in Stages 2, 3, and 5; however, it was not present in Stage 4. Therefore, crystallin αA might be involved in very early stages of cataract formation and later stages of severely opaque lenses. Very similar αB expression was noted in Stages 2 and 5.^28–32^

n-acetylcarnosine or carcinine eye drops resistant to enzymatic hydrolysis could act as pharmacological chaperones and decrease oxidative stress and excessive glycation in stress-related eyes such as cataracts. Ischemic diabetic retinopathy might protect against nuclear sclerotic cataracts, and these findings were consistent with the hypothesis that increased exposure to oxygen is responsible for nuclear cataract formation.^33^ None of the patients in this study used these drugs regularly, but the relations of these drugs’ antioxidative function and the crystalline changes noted in this study are worthy of advanced studies.^34^ In contrast, 2 well-known drugs, corticosteroids^35^ and the antipsychotic drug quetiapine,^36^ can induce cataracts; none of our patients received these drugs for >1 month; therefore, they were not the issues of our study. Cataracts are also classified by their location, with the posterior type usually due to steroid and diabetes mellitus.^35,37^ To decrease the special cataract type–induced bias in the study, the score of LOCS classification focus in any part, and the difference of any 2 parts over 4 (denoted as 0 and 4 by the 2 parts), were excluded from this study. To study the crystalline expression of different cataracts is also an important issue and worthy of study in the future. Other special type cataracts, such as traumatic cataracts, congenital cataracts, and exfoliation syndrome, were excluded in this study to obtain the simple information of natural progressing cataracts. In conclusion, crystallin protein levels and post-translational modifications were changeable during the progression of cataracts.

## Conclusions

Crystallin protein levels and post-translational modifications were changeable during the progression of cataracts. Understanding these protein dynamics during the formation of cataracts might help in designing distinct treatments for this disease.

## Conflicts of Interest

The authors have indicated that they have no conflicts of interest regarding the content of this article.

## Figures and Tables

**Table I:** Proteins identified from mass spectrometry.

Spot No.	Accession No.	Protein Identification	MW (kDa) /pI	Score	Post-translational Modification
1	P02489	α-Crystallin A chain	19.9/5.77	448	Ac Pi
2	P02489	α-Crystallin A chain	19.9/5.77	464	Ac Pi
3	P02489	α-Crystallin A chain	19.9/5.77	511	Ac Pi
4	P02489	α-Crystallin A chain	19.9/5.77	375	Pi
5	P02489	α-Crystallin A chain	19.9/5.77	561	Ac Pi
6	P02489	α-Crystallin A chain	19.9/5.77	642	Ac Pi
7	P02489	α-Crystallin A chain	19.9/5.77	383	Pi
8	P02489	α-Crystallin A chain	19.9/5.77	365	Ac
9	P53673	β-Crystallin A4	22.2/5.82	526	Pi
10	P53673	β-Crystallin A4	22.2/5.82	407	Pi
11	P53673	β-Crystallin A4	22.2/5.82	510	
12	P53673	β-Crystallin A4	22.2/5.82	546	Pi
13	P02489	α-Crystallin A chain	19.9/5.77	475	
14	P53673	β-Crystallin A4	22.2/5.82	413	
15	P53674	β-Crystallin B1	27.9/8.59	1068	Pi
16	P05813	β-Crystallin A3	25.1/5.81	665	Ac Pi
17	P53673	β-Crystallin A4	22.2/5.82	541	
18	P07320	γ-Crystallin D	20.6/7.15	519	
19	P53674	β-Crystallin B1	27.9/8.59	1077	Ac Pi
20	P53674	β-Crystallin B1	27.9/8.59	928	
21	P53674	β-Crystallin B1	27.9/8.59	1177	Pi
22	P53674	β-Crystallin B1	27.9/8.59	1145	
23	P53674	β-Crystallin B1	27.9/8.59	1073	
24	P53674	β-Crystallin B1	27.9/8.59	1215	Pi
25	P53674	β-Crystallin B1	27.9/8.59	1135	Pi
26	P53674	β-Crystallin B1	27.9/8.59	1081	Pi
27	P53674	β-Crystallin B1	27.9/8.59	932	
28	P53674	β-Crystallin B1	27.9/8.59	926	
29	P43320	β-Crystallin B2	23.2/6.54	679	
30	P07315	γ-Crystallin C	20.7/7.04	650	
31	P07320	γ-Crystallin D	20.6/7.15	450	
32	P07315	γ-Crystallin C	20.7/7.04	665	
33	P02511	α-Crystallin B chain	20.1/6.76	515	
34	P02511	α-Crystallin B chain	20.1/6.76	607	Pi
35	P02511	α-Crystallin B chain	20.1/6.76	591	Pi
36	P02511	α-Crystallin B chain	20.1/6.76	694	Pi
37	P02511	α-Crystallin B chain	20.1/6.76	565	
38	P02511	α-Crystallin B chain	20.1/6.76	407	
39	P02511	α-Crystallin B chain	20.1/6.76	338	
40	P02489	α-Crystallin A chain	19.9/5.77	409	Ac
41	Q01469	Fatty acid-binding protein	15.0/6.84	207	
42	P02489	α-Crystallin A chain	19.9/5.77	441	
43	P02489	α-Crystallin A chain	19.9/5.77	309	
44	P02489	α-Crystallin A chain	19.9/5.77	402	
45	P02489	α-Crystallin A chain	19.9/5.77	354	Ac Pi
46	P02489	α-Crystallin A chain	19.9/5.77	481	Ac Pi
47	P02489	α-Crystallin A chain	19.9/5.77	557	Ac Pi
48	P02489	α-Crystallin A chain	19.9/5.77	661	Ac Pi
49	P22914	β-Crystallin S	20.9/6.43	515	Ac Pi
50	P53674	β-Crystallin B1	27.9/8.59	586	
51	P02489	α-Crystallin A chain	19.9/5.77	597	Ac Pi
52	P02489	α-Crystallin A chain	19.9/5.77	564	Ac Pi
53	P05813	β-Crystallin A3	25.1/5.81	569	Pi
54	P05813	β-Crystallin A3	25.1/5.81	708	Ac Pi
55	P53674	β-Crystallin B1	27.9/8.59	1105	Ac Pi
56	P05813	β-Crystallin A3	25.1/5.81	753	Pi
57	P53674	β-Crystallin B1	27.9/8.59	1051	Ac Pi
58	P05813	β-Crystallin A3	25.1/5.81	688	Pi
59	P05813	β-Crystallin A3	25.1/5.81	847	Pi
60	P02511	α-Crystallin B chain	20.1/6.76	595	Pi
61	P43320	β-Crystallin B2	23.2/6.54	437	Ac Pi
62	P53674	β-Crystallin B1	27.9/8.59	1033	Pi
63	P53674	β-Crystallin B1	27.9/8.59	637	
64	P53674	β-Crystallin B1	27.9/8.59	1022	Pi
65	P53674	β-Crystallin B1	27.9/8.59	1082	Pi
66	P43320	β-Crystallin B2	23.2/6.54	703	
67	P05813	β-Crystallin A3	25.1/5.81	640	
68	P53674	β-Crystallin B1	27.9/8.59	1058	Pi
69	P53674	β-Crystallin B1	27.9/8.59	1366	Pi
70	P02511	α-Crystallin B chain	20.1/6.76	791	
71	P02511	α-Crystallin B chain	20.1/6.76	497	
72	P02511	α-Crystallin B chain	20.1/6.76	510	
73	P02511	α-Crystallin B chain	20.1/6.76	466	Pi
74	P05813	β-Crystallin A3	25.1/5.81	427	
75	P07320	γ-Crystallin D	20.6/7.15	216	
76	P02511	α-Crystallin B chain	20.1/6.76	239	
77	P02489	α-Crystallin A chain	19.9/5.77	373	
78	P53673	β-Crystallin A4	22.2/5.82	484	
79	P02489	α-Crystallin A chain	19.9/5.77	511	Ac Pi
80	P53673	β-Crystallin A4	22.2/5.82	442	
81	P53673	β-Crystallin A4	22.2/5.82	523	
82	P53673	β-Crystallin A4	22.2/5.82	478	
83	P53673	β-Crystallin A4	22.2/5.82	436	
84	P53673	β-Crystallin A4	22.2/5.82	362	
86	P02489	α-Crystallin A chain	19.9/5.77	364	Ac
87	P02489	α-Crystallin A chain	19.9/5.77	309	
88	P02489	α-Crystallin A chain	19.9/5.77	278	
89	P02489	α-Crystallin A chain	19.9/5.77	201	
90	P02489	α-Crystallin A chain	19.9/5.77	234	
91	P02489	α-Crystallin A chain	19.9/5.77	247	
92	P02489	α-Crystallin A chain	19.9/5.77	135	
93	P53674	β-Crystallin B1	27.9/8.59	155	
94	P53674	β-Crystallin B1	27.9/8.59	432	
95	P53674	β-Crystallin B1	27.9/8.59	718	
96	P53674	β-Crystallin B1	27.9/8.59	337	
97	P53674	β-Crystallin B1	27.9/8.59	945	
98	P53674	β-Crystallin B1	27.9/8.59	889	
99	P53674	β-Crystallin B1	27.9/8.59	1009	
100	P05813	β-Crystallin A3	25.1/5.81	691	
101	P05813	β-Crystallin A3	25.1/5.81	551	Ac Pi
102	P53674	β-Crystallin B1	27.9/8.59	1071	Pi
103	P53674	β-Crystallin B1	27.9/8.59	1029	
104	P05813	β-Crystallin A3	25.1/5.81	608	
105	P53674	β-Crystallin B1	27.9/8.59	1214	Pi
106	P05813	β-Crystallin A3	25.1/5.81	621	
107	P53674	β-Crystallin B1	27.9/8.59	786	
108	P53674	β-Crystallin B1	27.9/8.59	997	Pi
109	P53674	β-Crystallin B1	27.9/8.59	923	
110	P53674	β-Crystallin B1	27.9/8.59	1161	Pi
111	P53674	β-Crystallin B1	27.9/8.59	927	
112	P53674	β-Crystallin B1	27.9/8.59	876	
113	P05813	β-Crystallin A3	25.1/5.81	551	
114	P05813	β-Crystallin A3	25.1/5.81	475	
115	P07315	γ-Crystallin C	20.7/7.04	465	
116	P02511	α-Crystallin B chain	20.1/6.76	170	
117	P02511	α-Crystallin B chain	20.1/6.76	239	
118	P02489	α-Crystallin A chain	19.9/5.77	206	
119	P53673	β-Crystallin A4	22.2/5.82	192	
120	P53673	β-Crystallin A4	22.2/5.82	447	
121	P53673	β-Crystallin A4	22.2/5.82	539	
122	P02489	α-Crystallin A chain	19.9/5.77	296	
123	P53673	β-Crystallin A4	22.2/5.82	424	
124	P53673	β-Crystallin A4	22.2/5.82	475	
125	P53673	β-Crystallin A4	22.2/5.82	481	
126	P53673	β-Crystallin A4	22.2/5.82	444	
127	P53673	β-Crystallin A4	22.2/5.82	512	
128	P53673	β-Crystallin A4	22.2/5.82	396	
129	P02489	α-Crystallin A chain	19.9/5.77	485	
130	P53673	β-Crystallin A4	22.2/5.82	402	
131	P02489	α-Crystallin A chain	19.9/5.77	513	Ac Pi
132	P02489	α-Crystallin A chain	19.9/5.77	494	Ac Pi
133	P02489	α-Crystallin A chain	19.9/5.77	641	Ac Pi
134	P02489	α-Crystallin A chain	19.9/5.77	707	Ac Pi
135	P02489	α-Crystallin A chain	19.9/5.77	506	Ac Pi
136	P53673	β-Crystallin A4	22.2/5.82	517	
137	P53673	β-Crystallin A4	22.2/5.82	570	
138	P02489	α-Crystallin A chain	19.9/5.77	579	Ac Pi
139	P53673	β-Crystallin A4	22.2/5.82	553	Pi
140	P02489	α-Crystallin A chain	19.9/5.77	460	Ac Pi
141	P02489	α-Crystallin A chain	19.9/5.77	409	Ac Pi
142	P02489	α-Crystallin A chain	19.9/5.77	329	
143	P02489	α-Crystallin A chain	19.9/5.77	553	
144	P02489	α-Crystallin A chain	19.9/5.77	755	Ac Pi
145	P02489	α-Crystallin A chain	19.9/5.77	641	Pi
146	P53673	β-Crystallin A4	22.2/5.82	372	
147	P02489	α-Crystallin A chain	19.9/5.77	308	
149	P53673	β-Crystallin A4	22.2/5.82	405	
150	P02489	α-Crystallin A chain	19.9/5.77	439	Pi
151	P07320	γ-Crystallin D	20.6/7.15	458	
152	P02511	α-Crystallin B chain	20.1/6.76	775	PiAc
153	P07320	γ-Crystallin D	20.6/7.15	445	
154	P02511	α-Crystallin B chain	20.1/6.76	882	Pi
155	P02511	α-Crystallin B chain	20.1/6.76	948	PiAc
156	P43320	β-Crystallin B2	23.2/6.54	1292	Ac
157	P43320	β-Crystallin B2	23.2/6.54	1346	PiAc
158	P02511	α-Crystallin B chain	20.1/6.76	693	
159	P02511	α-Crystallin B chain	20.1/6.76	489	
160	P02511	α-Crystallin B chain	20.1/6.76	895	PiAc
161	P02511	α-Crystallin B chain	20.1/6.76	982	PiAc
162	P02511	α-Crystallin B chain	20.1/6.76	964	Pi
163	P02511	α-Crystallin B chain	20.1/6.76	268	
164	P02511	α-Crystallin B chain	20.1/6.76	228	Pi
166	P02511	α-Crystallin B chain	20.1/6.76	758	
167	P02511	α-Crystallin B chain	20.1/6.76	821	Pi
169	P02511	α-Crystallin B chain	20.1/6.76	282	
170	P02511	α-Crystallin B chain	20.1/6.76	307	
171	P02489	α-Crystallin A chain	19.9/5.77	314	
172	P02489	α-Crystallin A chain	19.9/5.77	255	Ac
173	P02489	α-Crystallin A chain	19.9/5.77	284	
175	P02489	α-Crystallin A chain	19.9/5.77	143	

Ac, acetylation; MW, molecular weight; Pi, phosphorylation; PiAc, phosphorylation, acetylation; pI, isoelectric point.

**Table II:** The proteins expressed in cataracts.

Stage	Total Protein Number	Proteins (no.)					Phosphorylation (%)	Acetylation (%)	Truncation (%)
2	44	βB1 11	αB 11	αA 11	βA4 8	Others 3	19 (46.3)	8 (19.5)	19 (46.3)
3	47	αA 13	βB1 10	βA3 6	βA4 6	Others 12	19 (54.3)	10 (28.6)	25 (71.4)
4	38	βB1 16	βA4 9			Others 13	4 (16)	0	0
5	46	αA 19	βA4 14	αB 6		Others 7	20 (51.3)	4 (10.3)	19 (48.7)
